# The Effects of Light Crystal Display 3D Printers, Storage Time and Steam Sterilization on the Dimensional Stability of a Photopolymer Resin for Surgical Guides: An In Vitro Study

**DOI:** 10.3390/ma18030474

**Published:** 2025-01-21

**Authors:** Nicola Pranno, Alessio Franchina, Francesca De Angelis, Maurizio Bossù, Alessandro Salucci, Edoardo Brauner, Maria Paola Cristalli, Gerardo La Monaca

**Affiliations:** 1 Department of Oral and Maxillo-Facial Sciences, Sapienza, University of Rome, 6 Caserta Street, 00161 Rome, Italy; nicola.pranno@uniroma1.it (N.P.); francesca.deangelis@uniroma1.it (F.D.A.); maurizio.bossu@uniroma1.it (M.B.); alessandro.salucci@uniroma1.it (A.S.); edoardo.brauner@uniroma1.it (E.B.); mariapaola.cristalli@uniroma1.it (M.P.C.);; 2 Indipendent Researcher, 44 Legione Gallieno Street, 36100 Vicenza, Italy

**Keywords:** 3D printing, digital light processing, LCD technology, 3D surgical guide, photoactive resin, sterilization

## Abstract

Background: Implant surgical guides manufactured in-house using 3D printing technology are widely used in clinical practice to translate virtual planning to the operative field. Aim: The present in vitro study investigated the dimensional changes of 3D surgical guides printed in-house using Shining 3D surgical guide resin (SG01). Materials and methods: Five test bodies, varying in shape and dimensions, were designed using computer-aided design (CAD) software and manufactured using three different Light Crystal Display (LCD) 3D printers (AccuFab-L4D, Elegoo Mars Pro 3, and Zortrax Inspire). Specific printing and post-processing parameters for the SG01 resin were set to produce 25 test bodies (5 of each shape) from each of the three printers, resulting in a total of 75 samples. The dimensional changes were evaluated using a digital calliper at four different time points: immediately after printing (T0), one month after storage (T1), immediately after sterilization (T2), and one month after sterilization (T3). Results: All the test bodies showed deviations from the overall CAD reference value of 12.25 mm after printing and post-processing (T0) and following steam sterilization (T2). Similar trends were observed for the effect of storage times at T1 and T3. The AccuFab prints demonstrated a better dimensional stability than the Elegoo and Zortrax samples. Conclusions: The LCD 3D printers, sterilization, and storage times influenced the dimensional stability of the test bodies made with SGO1 resin.

## 1. Introduction

Computed tomography (CT) and computer-aided design/computer-assisted manufacture (CAD/CAM) technologies have significantly enhanced implant surgery. Cone beam computed tomography (CBCT) imaging, intraoral surface scanning, and three-dimensional (3D) surgical planning software have allowed clinicians to analyze the patient’s anatomic structures and prosthetic parameters and virtually plan the optimal implant position and direction [1,2,3,4].

Surgical guides are essential in this digital workflow as they transfer virtual planning to the operative field, improving the accuracy and predictability of implant placement [5,6]. Implant surgical guides can be produced by two major manufacturing techniques, subtractive and additive [7,8]. In the subtractive method, the guide is milled from a larger polymer blank using a computer-numeric-controlled machine [9]. In the additive approach, the guide is produced using 3D printing. This process involves sequential layering and curing each layer through three main different technologies: digital light processing, stereolithography, or fused filament fabrication [9,10]. Currently, additive manufacturing methods are more widely used than milling procedures due to their ability to create complex geometries, cost-effectiveness, and minimal waste generation [11,12,13,14]. Moreover, the widespread adoption of additive techniques has also been promoted by the commercialization of inexpensive 3D printers, which allow for in-house guide production, reduce the need for central manufacturing facilities, and improve availability [15,16].

Nevertheless, regardless of the technologies employed, the digital workflow for producing surgical guides affects their dimensional accuracy, which is essential for ensuring that the planned and actual positions of the implants match. The guides’ accuracy depends on some cumulative and interactive possible errors involving data acquisition, management, merging, dimensional changes due to the manufacturing process, guide stabilization, and bone characteristics [17].

Specifically, 3D printing technologies may lead to dimensional changes in the surgical guide, reducing accuracy and negatively affecting clinical outcomes. Dimensional changes are linked to photopolymerizing resins, which undergo chemical and physical modifications induced by processing and post-processing procedures and environment exposure [17].

Another critical aspect impacting the dimensional stability of 3D surgical guides is sterilization, which is needed to prevent infections and postoperative complications. Indeed, surgical guides, coming into contact with the patient’s blood, oral tissues, and exposed bone, are subject, like other medical devices, to the same safety standards set by the EU Medical Device Regulation guidelines and Centers for Disease Control and Prevention (CDC, USA) [18,19,20].

Numerous studies have recently examined how printing materials, processing and post-processing technology, and sterilization methods can affect potential changes in the dimensional stability of 3D surgical guides [8].

Knowing this information before the clinical use of surgical guides is important to understand their behaviour in surgical procedures and making informed decisions, especially in light of the increasing diffusion of in-house manufacturing. Therefore, the present in vitro study aimed to investigate the effect of Light Crystal Display (LCD) 3D printing technology, steam sterilization, and storage times on the dimensional stability of SGO1 polymer resin for manufacturing 3D surgical guides.

The null hypotheses were that the dimensional stability of the test bodies manufactured with the Shining 3D surgical guide resin (SG01) would not be significantly affected by the three tested variables: different LCD 3D printers, sterilization, and storage times.

## 2. Materials and Methods

The manuscript was drafted following the modified CONSORT checklist of items for reporting in vitro studies on dental materials [21].

### 2.1. Study Design

In this in vitro study, the Shining 3D surgical guide resin SG01 (chemical composition: phenyl bis (2,4,6-trimethylbenzoyl)-phosphine oxide, Polymeric urethane acrylate, 2-Propen-1-one, 1-(4-morpholinyl), (Octahydro-4,7-methano-1H-indenediyl)bis(methylene) diacrylate, Ethoxylated Bisphenol A, Poly[oxy(methyl-1,2-ethanediyl)], α,α′-(2,2-dimethyl-1,3-propanediyl)bis[ω-[(1-oxo-2-propen-1-yl)oxy]), was used as the printing material. This transparent, biocompatible photopolymer resin is certified by the China FDA as a Class I medical device and meets ISO 10993 standards. The resin is designed explicitly for 3D-printed customized dental models that are non-implantable and can be used for body contact for up to 24 h.

The experimentation involved three LCD 3D printers: AccuFab-L4D from Shining 3D Tech. Co., Ltd. (Hangzhou, China), Elegoo Mars Pro 3 from Elegoo Technology Co., Ltd. (Shenzhen, China), and Zortrax Inspire from Zortrax S.A. (Olsztyn, Poland), as outlined in Table 1. The rationale for selecting the printers was routine use by the authors for in-house printing of surgical guides, intending to eliminate any bias due to the operators’ inexperience.

Test bodies were used as a reference because they are the simplest benchmark objects for evaluating the dimensional stability of 3D printing resin [22].

The study protocol involved manufacturing 5 different test body designs, each printed 5 times using three LCD 3D printers (total = 75), and evaluating dimensional changes at four different time points: immediately after printing and post-processing (T0), one month after (T1), immediately after sterilization (T2), and one month after (T3).

The choice of a one-month timeframe after post-processing and after sterilization was based on previous in vitro studies, which suggested stabilizing dimensional changes after this period [23,24].

### 2.2. Test Body Manufacture

**Figure 1:**
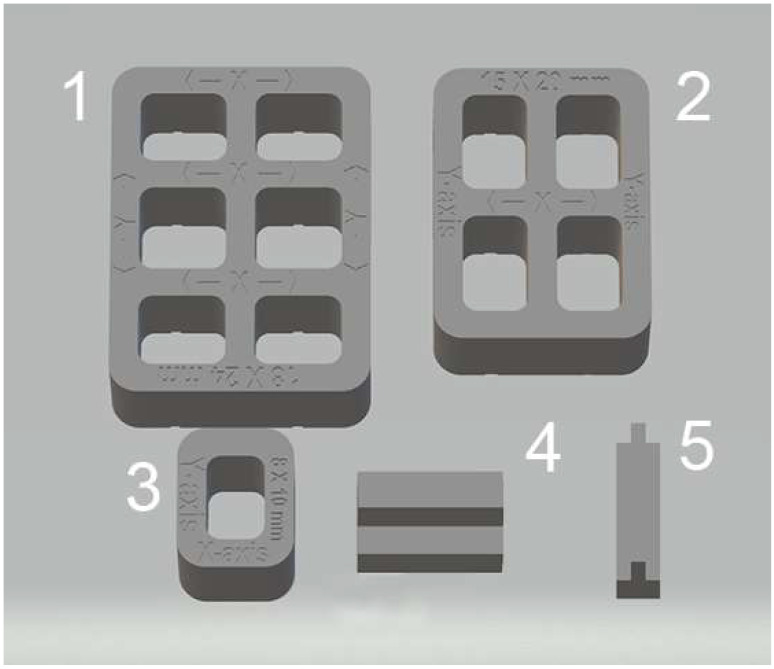
Shapes and CAD reference values in mm of test body designs: (**1**) x-axis = 18, y-axis = 24; (**2**) x-axis = 15, y-axis = 20; (**3**) x-axis = 8, y-axis = 10; (**4**) thickness = 2; (**5**) thickness = 1.

Test bodies were designed in five different geometric shapes and dimensions using a computer-aided design (CAD) software program (SolidWorks 3D CAD, Dassault Systèmes, Vélizy-Villacoublay, France) to replicate implant surgical guide characteristics (Figure 1).

The CAD file of each test body’s design was exported as a standard tessellation language (STL) file format and then imported into the respective slicing software of the three 3D printers selected to manufacture 5 samples from each device. A total of 75 test bodies were produced, 25 for each printer, split into 5 samples for each of the 5 designs. The printing parameters were the same for all three devices. The specific parameters for Shining 3D surgical guide resin SG01 (100 µm layer printing and 405 nm wavelength) were set to optimize the resin’s curing process and prevent warping or shrinkage. The build platform, fixed at a 0° orientation without additional support structures, was programmed to minimize potential deformation due to uneven thermal or mechanical stresses during printing and support removal. Each test body was labelled for future identification using a letter for the printer and a number for the design.

After 3D printing, all 75 test bodies underwent post-processing using the Shining 3D FabWash system (Shining 3D Tech. Co., Ltd., Hangzhou, China) to remove any uncured resin from the print surface, and Shining3D FabCure 2 Post-curing unit (Shining 3D Tech. Co., Ltd., Hangzhou, China) to maximize the material’s mechanical properties. The washing was an automatic process involving a 6 min bath in 90% isopropyl alcohol (IPA) and air drying overnight. For post-curing, each side was exposed to a powerful UV light emitted by 30 multi-directional LEDs for 5 min, as recommended by the resin manufacturer.

Following post-processing, test bodies were measured, sealed in separate plastic bags, and stored at room temperature. One month later, they were measured again, placed in separate sealed sterilization pouches, and autoclaved (Melag-Vacuklav^®^ 40 B+ evolution, Berlin, Germany) at 134 °C and 2 bar for 10 min. The steam sterilization method was chosen because it is widely used in medical and dental practices and effectively ensures sterility without introducing chemical residues.

### 2.3. Dimension Assessment

Test bodies were measured manually at four different time points using a digital calliper (Mitutoyo America, Aurora, IL, USA) with an accuracy of ±0.03 mm and a resolution of 0.01 mm. All measurements were taken in triplicate by two independent blinded examiners, and the mean values were utilized to evaluate dimensional changes. Before the study, to reduce the risk of errors, the examiners underwent calibration and training sessions to ensure the consistent use of the digital calliper.

Dimensional changes were assessed after the 3D printing and post-processing at T0 and following sterilization at T2. Measures at T1 and T3 referred to the time frames typically elapsing between production and sterilization and between sterilization and the intra-operative use of the surgical guides, respectively.

Measurements of the linear dimensions (distances between fixed points) and thicknesses taken of test bodies were compared to the known standard dimensions of the five virtual designs. The CAD reference values for the five test bodies (Figure 1) were as follows: 18 mm (x-axis) and 24 mm (y-axis) for sample 1; 15 mm (x-axis) and 20 mm (y-axis) for sample 2; 8 mm (x-axis) and 10 mm (y-axis) for sample 3; 2 mm thickness (z-axis) for sample 4; 1 mm thickness (z-axis) for sample 5. The mean of these eight CAD reference values resulted in an overall CAD reference value of 12.25 mm. Identifying nine measurements allowed an accurate dimensional evaluation of each group of test bodies.

### 2.4. Statistical Analysis

The required sample size was calculated using GPower statistical software (version 3.1.9.2, Heinrich-Heine-Universität, Düsseldorf, Germany). A power analysis for repeated measures ANOVA with between-factor effects was conducted using an alpha level of 0.05, an effect size of 0.43, and a power of 0.95 (95%). This calculation determined that a minimum of 64 samples would be needed to detect statistically significant differences. The power calculation was based on the overall value derived from the first two groups of five samples for each printer at different time points.

Statistical analysis was performed using SPSS version 27.0 (IBM Corporation, Armonk, New York, NY, USA). Descriptive statistics are presented as means (±standard deviations). The Shapiro–Wilk test was applied to assess the normality of the data distribution. Box plots were used to identify any potential outliers within the data. A two-way ANOVA was conducted on normally distributed data to assess the effects of printer type and time variable (T0, T1, T2, and T3) on dimensional stability. Post hoc pairwise comparisons were performed using Bonferroni adjustment where necessary. Intraclass Correlation Coefficients (ICCs) were calculated to assess intra-operator and inter-operator reliability. Reliability was evaluated using repeated measurements by two investigators at two and three weeks after the initial measurements. Values greater than 0.90 were considered excellent, 0.75–0.90 as good, 0.50–0.75 as moderate, and below 0.50 as poor. A significance level of *p* ≤ 0.05 was set for all statistical tests.

## 3. Results

All 75 manufactured test bodies were successfully included in the analysis, with no exclusions due to defects, incomplete curing, or measurement errors. The dataset detailing the mean value of measurements for all test bodies at different time points (T0, T1, T2, and T3) is reported in Table 2.

The intra-operator reliability for the first examiner yielded a Cronbach’s alpha of 1.000 and an ICC of 1.000 for both single and mean measures, indicating the complete consistency in the measurements taken by the same operator over time (95% CI [1.000, 1.000], F (39, 39) = 2,135,929.244, *p* < 0.001).

The inter-operator reliability between the two examiners showed strong agreement, with a Cronbach’s alpha of 1.000, an ICC of 0.999 for the single measures, and 1.000 for the mean measures (95% CI [0.999, 1.000], F (39, 39) = 2791.201, *p* < 0.001). These findings confirm a high degree of precision and consistency in the dimensional measurements across all the test conditions.

### 3.1. Effect of Printing and Post-Processing (T0)

**Figure 2:**
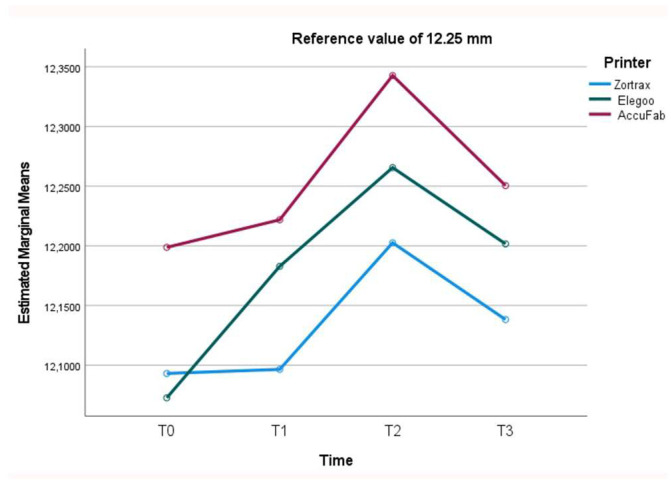
Line graph describing overall dimensional changes (mm) of test bodies for each 3D printer and for each time point (T0, T1, T2, and T3).

**Figure 3:**
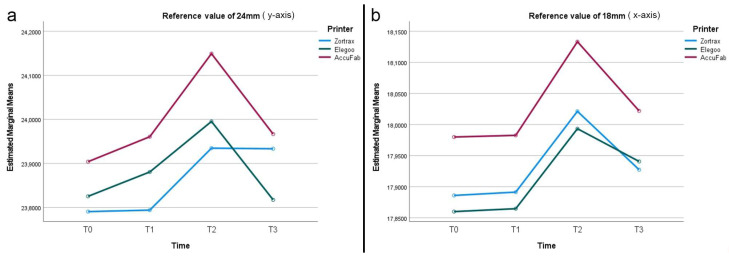
Line graphs describing dimensional changes (mm) of test bodies (virtual design 1): (**a**) y-axis; (**b**) x-axis.

**Figure 4:**
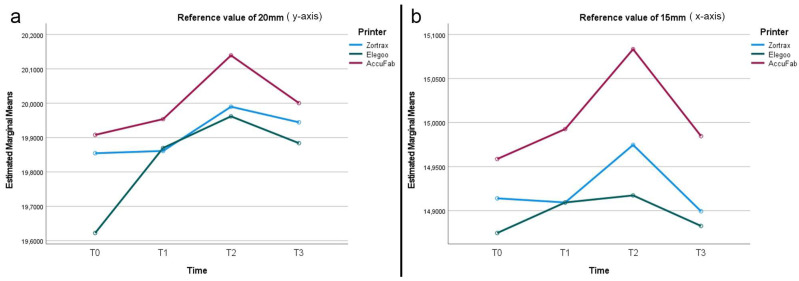
Line graphs describing dimensional changes (mm) of test bodies (virtual design 2): (**a**) y-axis; (**b**) x-axis.

**Figure 5:**
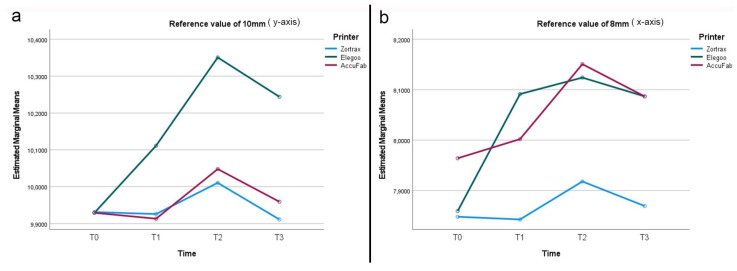
Line graphs describing dimensional changes (mm) of test bodies (virtual design 3): (**a**) y-axis; (**b**) x-axis.

**Figure 6:**
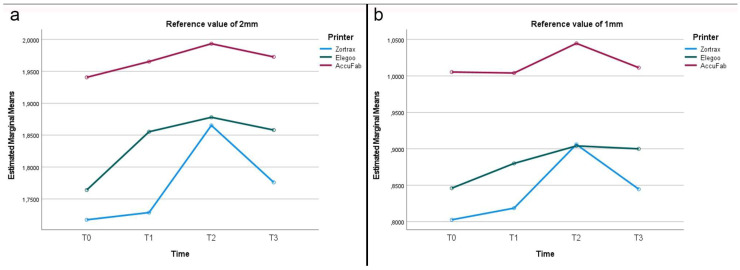
Line graphs describing dimensional changes (mm) in the thickness of test bodies: (**a**) virtual design 4; (**b**) virtual design 5.

After printing and post-processing, the AccuFab test bodies demonstrated the best dimensional stability, followed by those from the Elegoo and Zortrax printers, with deviations of −0.051 mm, −0.157 mm, and −0.177 mm from the overall CAD reference of 12.25 mm, respectively (Figure 2). The higher accuracy of the AccuFab prints compared to that of the samples from the other two printers was also confirmed by the x- and y- axes and the thickness measurements. For virtual design 1 (Figure 3), having the largest dimensions, the deviations from the individual CAD reference values (y-axis = 24 mm, x-axis = 18 mm) were −0.096 mm (y-axis) and −0.020 mm (x-axis) for the AccuFab test bodies; −0.209 mm (y-axis) and −0.114 mm (x-axis) for Elegoo test bodies; and −0.175 mm (y-axis) and −0.122 mm (x-axis) for the Zortrax test bodies. Similar results were observed for the intermediate-sized design (Virtual Design 2). The deviations along the y-axis were as follows: −0.092 mm, −0.145 mm, and −0.377 mm for the AccuFab, Elegoo, and Zortrax printers, respectively. For the x-axis, the deviations were −0.041 mm, −0.086 mm, and −0.125 mm, also corresponding to the AccuFab, Elegoo, and Zortrax printers, respectively (Figure 4). For virtual design 3 (Figure 5), which had the smallest dimensions, the deviations from the individual CAD reference values (y-axis = 10 mm, x-axis = 8 mm) were as follows: for the AccuFab specimens, −0.071 mm (y-axis) and −0.036 mm (x-axis); for the Elegoo specimens, −0.056 mm (y-axis) and −0.021 mm (x-axis); for the Zortrax specimens, −0.062 mm (y-axis) and −0.022 mm (x-axis). For virtual design 4 (Figure 6), the increased thickness further supported the AccuFab printer’s superiority over Elegoo and Zortrax, with a deviation from the individual CAD reference (2.0 mm) of −0.059 mm, −0.533 mm, and −0.482 mm, respectively.

### 3.2. Effect of Storage Time After Printing and Post-Processing (T1)

After 1 month of storage after printing and post-processing, slight dimensional changes were observed in all the test bodies (Figure 2). The deviations measured from the overall CAD reference of 12.25 mm were −0.028 mm for the samples printed by AccuFab, −0.154 mm for those by Elegoo, and −0.067 mm for those by Zortrax. Compared to the individual reference values of the larger sample (y-axis = 24 mm, x-axis = 18 mm) (Figure 3), the AccuFab prints showed minimal deviations (y-axis = +0.011 mm, x-axis = +0.007 mm) than the prints by Elegoo (y-axis= −0.039 mm, x-axis = −0.109 mm) and Zortrax (y-axis = −0.119 mm, x-axis = −0.135 mm). Additionally, the measurements of the smallest-sized sample (y-axis = 10 mm, x-axis = 8 mm) (Figure 5) confirmed that the test bodies printed with AccuFab displayed greater resilience to dimensional changes over the storage period, with deviations of −0.087 mm on the y-axis and −0.063 mm on the x-axis compared to those printed by Eligoo (y-axis= 0.074 mm, x-axis = 0.079 mm) and by Zortrax (y-axis = −0.082 mm, x-axis = −0.091). The thickness evaluation of sample 4 (Figure 6) confirmed better dimensional stability for the AccuFab specimens than those printed by Elegoo and Zortrax, with deviations from the individual CAD references of −0.035 mm, −0.131 mm, and −0.115 mm, respectively.

### 3.3. Effect of Steam Sterilization (T2)

The impact of steam sterilization on the dimensional changes showed significant variations from the overall CAD reference value (12.25 mm) in all the test bodies, with a slight expansion trend (Figure 2). The AccuFab prints displayed a minor deviation (+0.093 mm) compared to the samples manufactured by the Elegoo (+0.047 mm) and Zortrax (+0.116 mm) printers. The analysis of the individual measures revealed that steam sterilization led to more pronounced dimensional changes in the smaller test bodies, particularly for Zortrax and Elegoo. For virtual design 3 (y-axis = 10 mm, x-axis = 8 mm) (Figure 5), the smallest deviations were observed for AccuFab (y-axis= +0.098 mm, x-axis = +0.151 mm), followed by Elegoo (y-axis = +0.089 mm, x-axis = +0.126 mm), and Zortrax (y-axis = +0.113 mm, x-axis= +0.134 mm). In sample 4 (CAD reference = 2.0 mm) (Figure 6), the test bodies printed by AccuFab, Elegoo, and Zortrax exhibited thickness deviations of +0.052 mm, +0.105 mm, and +0.083 mm, respectively.

### 3.4. Effect of Storage Time After Steam Sterilization (T3)

After an additional month of storage following sterilization, compared to the overall CAD reference value (12.25 mm), no dimensional changes in the AccuFab specimens and minimal dimensional recovery of the samples from Elegoo (−0.067 mm) and Zortrax (−0.083 mm) were detected (Figure 2). The individual measurements of the largest test bodies (virtual design 1) (Figure 3) displayed slight deviations from those printed by AccuFab (y-axis = −0.033 mm, x-axis = −0.009 mm), Elegoo (y-axis = −0.055 mm, x-axis = −0.012 mm), and Zortrax (y-axis = −0.112 mm, x-axis = −0.017 mm), suggesting a compounded effect of storage and sterilization. The dimensional stability with minimal cumulative effects over time was validated by an assessment of the smaller-sized test bodies (virtual design 3) and thickness (virtual design 4). For the smaller-dimensional specimens (Figure 5), deviations from the individual CAD reference (y-axis = 10 mm, x-axis = 8 mm) were y-axis = −0.041 mm and x-axis = −0.013 mm for those printed by AccuFab; y-axis = −0.089 mm and x-axis = −0.131 mm for the Elegoo samples; and y-axis = −0.091 mm and x-axis = −0.087 mm for the Zortrax samples. For thickness (Figure 6), the deviations were +0.013 mm for the AccuFab prints, +0.057 mm for the Elegoo prints, and +0.040 mm for the Zortrax prints. According to these results, prolonged storage post-sterilization seemed to have a negligible impact on the dimensional stability of all the test specimens, with those printed by AccuFab showing better performance.

### 3.5. Effect of Printer Type and Time (T0, T1, T2, and T3) Variable

The combined analysis of the printer type (*p*-value = 0.034) and time variable (*p*-value = 0.020) significantly influenced the dimensional changes of all the test bodies. The samples printed by the AccuFab device achieved a better accuracy than those produced by Elegoo and Zortrax across all four time points, with the highest value at TO.

The effect of the time variable was particularly notable at T2 and less significant at T1 and T3, suggesting that storage alone (before or after sterilization) had a negligible impact on the dimensional stability compared to heat and moisture during sterilization. The interaction between the printer type and time variable was not statistically significant (*p*-value = 0.765), indicating that the differences between the test bodies from the different printers were consistent at all four time points.

## 4. Discussion

The null hypotheses were rejected based on the statistical analysis of the results, which confirmed that the three tested variables—different LCD 3D printers, sterilization, and storage times—affected the dimensional stability of the test bodies manufactured with the SGO1 polymer resin.

All the test bodies showed deviations from the overall CAD reference value of 12.25 mm after printing and post-processing (T0) and following steam sterilization (T2). A similar trend was observed for the effect of storage times at T1 and T3. The AccuFab prints demonstrated better dimensional stability than the Elegoo and Zortrax samples.

To the best of the authors’ knowledge, this is the only study that has evaluated the dimensional stability of SG01 resin used for printing surgical guides with LCD 3D technology. Therefore, comparing the current findings with previous in vitro reports on the accuracy of 3D-printed surgical guides has been challenging due to differences in study designs, 3D printers, printing materials, printing and post-processing parameters, and evaluation methodologies.

### 4.1. Effect of Printing and Post-Processing (T0)

Several studies have explored how different additive manufacturing technologies, 3D printers, and resin materials influence the accuracy of surgical guides, reporting slightly contrasting results [6,24,25,26].

In in vitro research, Chen et al. evaluated the dimensional changes of CAD-CAM surgical guides produced using three different materials and additive manufacturing technologies immediately after production and after a one-month storage period [23]. They showed that combinations of various materials and 3D printers influenced the surgical templates’ accuracy, reproducibility, and stability. However, the absolute values of all dimensional differences, even if statistically significant, were small and may not cause any clinically notable effects.

A clinically acceptable accuracy between the definitive and planned implant position was also shown in the in vitro study conducted by Herschdorfer et al. for assessing the effect of various additive manufacturing technologies on the fabrication of surgical templates [27].

Wegmüller et al. reached similar conclusions when they compared consumer-level desktop 3D printers and high-end professional 3D models [15]. They found statistically significant differences in producing drill guides but negligible deviations from a clinical point of view. In the authors’ opinion, desktop 3D printers can produce drill guides with an accuracy comparable to that of professional models and at reduced costs.

Recently, Bathija et al., in an in vitro study, compared the accuracy of CAD-CAM surgical templates fabricated using five different additive manufacturing technologies by evaluating the final implant position against the initial digital implant plan [28]. Despite significant implant deviations between the different printing technologies, the results for all groups were still clinically acceptable because they were within the 2 mm safety margins of the mesial, distal, buccal, lingual, and apicocoronal limits.

### 4.2. Effect of Storage Time After Printing and Post-Processing (T1)

The dimensional changes over time of 3D printing photopolymerizing resin materials and the corresponding effect on the accuracy of 3D printed surgical guides are not fully understood. Only two studies [23,24] have reported data on surface variations after the post-production stage, which were difficult to compare with the current findings on the effect of storage time after printing and post-processing on the dimensional stability of the test bodies, due to the differences in the assessment methodologies and 3D printing technologies. In the study by Chen et al., reduced accuracy was found in resin surgical templates produced by a laboratory-based PolyJet 3D printer and a desktop SLA 3D printer [23]. In an in vitro investigation on the dimensional stability of surgical guides printed using DLP processing technology over 0, 5, 10, and 20 days, Lo Russo et al. showed that the time after manufacturing, alone or nested with guide volume, influenced the template’s dimensional variability, although the variations were limited [24].

### 4.3. Effect of Steam Sterilization (T2)

Another critical aspect that may lead to inaccuracies in implant insertion is the impact of steam sterilization on the guides’ dimensional changes and material biomechanical properties. Conflicting results have been reported in studies that have explored the effects of autoclaving at different cycles on samples manufactured using various printing technologies and resin materials.

Labakoum et al. recently assessed the effects of steam sterilization at 121 °C, +1 bar for 20 min and at 134 °C, + 2 bar for 10 min on the dimensional and mechanical characteristics of 3D surgical guides made with a procedure similar to that adopted in the present study [29]. The samples were printed using LCD technology (Mars 2 PRO printer -Elegoo, Shenzhen, China) and SG100 resin, immersed in 99% isopropanol alcohol for 5 min, air-dried, and then subjected to photopolymerization at a wavelength of 405 nm and temperature of 25 °C for 10 min. The results indicated that the mechanical and geometric characteristics were influenced by steam sterilization. After being autoclaved at 121 °C, the samples showed a decreased tensile load and deformation during bending. However, when autoclaved at 134 °C, the maximum compressive load and compressive strength increased significantly; standard tensile tests revealed only slight deformation and the findings from flexion evaluations remained unchanged.

Autoclaving-induced dimensional changes were also found by Hüfner et al., investigating 3D surgical guides made from different resin/printer combinations. All samples, except for the E-Guide/Micro Plus XL (Korg, Via Cagiata, 85, 60027 Campocavallo AN, Italy) combination, experienced minor shrinkage, which varied in all dimensions [30]. Furthermore, changes differed significantly among the materials following autoclaving with a cycle at 121 °C, whereas no significant difference was shown between the groups when a cycle at 134 °C was employed.

In a similar study on 3D-printed surgical templates from five different materials, Yazigi et al. evaluated the accuracy after sterilization in an autoclave at 121° C and a pressure of 2 bar for 20 min [31]. Significant dimensional changes in increased vertical discrepancy and angulation levels were detected for all the materials. However, these changes were still acceptable when an additional load was applied during the repositioning of the guides onto test models. Moreover, the choice of material significantly impacted the measured vertical discrepancies but did not affect the angulation measurements.

Mean and labial deformations and changes in the axial position of implants were reported by Li et al. in surgical guides printed using DLP technology, autoclaved at 134 °C for 5 min, and dried for 15 min [32]. Based on their findings, the authors concluded that this sterilization method was unsuitable for the clinical treatment of surgical guides.

The effects of disinfection and autoclave sterilization on the mechanical properties of surgical guides made using SLA and DLP printing were investigated by POP et al. [33] Regarding the DLP printing method, templates autoclave-sterilized at 121 °C, +1 bar, for 20 min showed statistically significant increases in the flexural strength and flexural modulus values. Compared to those autoclave-sterilized at 134 °C, +2 bar, for 10 min, these specimens demonstrated a significant increase in flexural strength and flexural strain and a decrease in the flexural modulus. Conversely, the tensile strength, tensile modulus, and tensile strain values were decreased in the templates sterilized at 134 °C rather than those autoclaved at 121 °C. It was not only the temperature value that influenced the mechanical behaviour but also the exposure time of the materials at that temperature because a more extended duration of exposure increases the material stiffness. In the authors’ opinion, the increased rigidity of the material could have implications in the clinical setting, making the guides more likely to fracture when pressure against the dental arches during surgery subjects them to combined loading (compression accompanied by bending).

Kirschner et al. studied how steam sterilization influenced the Vickers hardness and flexural modulus of insertion guides 3D-printed with five different methods and resin materials [34]. The results indicated that the autoclaving cycles led to more pronounced changes in the Vickers hardness and a minor impact on the flexural modulus (lower for all specimens). The variations observed depended on the specific resin and printer combination. The authors concluded that steam sterilization could alter the mechanical properties of the templates to some extent, especially with a cycle at 134 °C.

Unlike the results mentioned above, some studies found no significant effects of steam sterilization on the accuracy of 3D-printed surgical guides.

In a pilot study by Marei et al., the impact of steam heat sterilization at 121 °C for 20 min on the dimensional stability of surgical guides 3D-printed both in-office and in the laboratory using Class I biocompatible resin material was examined [35]. The findings showed no significant impact on the dimensional changes of the tested parts and no statistical differences between the in-office and laboratory 3D-printed samples.

Similar conclusions were reached by Sharma et al., who deemed not clinically relevant the overall linear expansion observed in test bodies manufactured in-house using PolyJet Glossy and SLA-LT printers and five different Class IIa biocompatible resins (proprietary and third-party) [22]. Additionally, even though statistically significant differences were noted among the two resin groups, the dimensional accuracy of the test bodies fabricated with the third-party resins was within a comparable range to that of those fabricated using the proprietary materials. This finding could support the use of support third-party resins as a cost-effective alternative for in-house 3D printing setups.

Likewise, Török et al. did not show any measurable deformation or structural change in 3D-printed surgical guides made with PolyJet technology after sterilization both at 121 °C for 20 min under a pressure of 1 bar and at 134 °C for 10 min under a pressure of 2 bar [36]. The only exception was the significant difference in the hardness strength of the autoclave-sterilized specimens at 134 °C. According to these results, the authors considered low- and high-temperature sterilization an appropriate method for sterilizing 3D-printed dental implant drill guide templates.

### 4.4. Effect of Storage Time After Steam Sterilization (T3)

The present findings regarding the negligible impact of the storage time after steam sterilization on the dimensional stability of the test bodies did not find a match in the literature, as these data have not been reported in similar investigations. Nevertheless, this information is helpful because surgical guides are not always used immediately after sterilization.

### 4.5. Effect of Printer Type and Time (T0, T1, T2, and T3) Variable

This study used a combined printer type and time variable analysis to provide easy-to-interpret summary data on the greater dimensional stability of samples printed by the AccuFab device compared to those produced by Elegoo and Zortrax across all four time points, with the highest value at TO.

### 4.6. Limitations and Strengths

The present study presents some limitations. The first was the difference between the test bodies and surgical guides in terms of the geometric morphology, which is flat in the former and convex or concave, depending on the shape of the jaws, in the latter. Additionally, the absence of metal sleeves could impact dimensional changes, as adding sleeves causes extra tension. However, recent studies have shown no significant differences between 3D-printed surgical guides with and without metal sleeves [37,38]. Furthermore, the resin biomechanical properties, such as the flexural strength and hardness, were not analyzed. Variations in these parameters potentially lead to fracturing and bending during surgical guide application, ultimately resulting in inaccuracies in implant insertion [33,34]. Another limitation is that the study only focused on one steam sterilization protocol and did not consider other chemical disinfection techniques or sterilization methods such as dry heat, ethylene oxide (ETO) gas, and hydrogen peroxide gas plasma. However, it is important to note that the chosen protocol is widely used in dental practices due to its simplicity and effectiveness.

The strength of this in vitro study lies in the protocol’s simplicity, reproducibility, and reliability. The test bodies are the simplest benchmark objects for evaluating the dimensional stability of the polymer-based guides 3D-printed in-house. The measurement method, performed with a digital calliper by two independent, blinded examiners, demonstrated excellent intra-operator and inter-operator reliability, confirming a high degree of precision and consistency in the dimensional measurements across all the test conditions.

## 5. Conclusions

Within the limitations of this in vitro study, printing with LCD technology, steam sterilization, and the storage times were shown to impact the dimensional stability of the test bodies manufactured in-house with Shining 3D Biocompatible Resin SG01. Specifically, the specimens produced by the AccuFab printer performed better than those made by the Elegoo and Zortrax devices. However, since the observed dimensional changes, although not statistically significant, could influence the clinical performance, the described workflow should be considered a new proposal for the in-house production of surgical guides that remains to be investigated. Therefore, further studies are needed to elucidate the influence of the Shining 3D Biocompatible Resin SG01 and LCD printing technologies on the accuracy of the surgical guides in transferring digital planning to the operative field. In vitro research and controlled clinical trials should focus on comparing the implant’s postoperative position to its digitally planned position.

## Figures and Tables

**Table 1:** LCD 3D printers: technical specifications.

Technical Specifications	Shining 3D AccuFab-L4D	Elegoo Mars Pro 3	Zortrax Inkspire
Resolution	3840 × 2400 (4K) Px	8520 × 4320	50 × 50 μm
Print Volume (x/y/z)	192 × 120 × 180 mm	153.36 × 77.76 × 175 mm	132 × 74 × 175 mm
Accuracy	±50 µm	50 µm	50 µm
Layer Thickness	25, 50, 75, 100 μm	50 µm	25, 50, 100 μm
Light Source	UV LED/LCD (wavelength 405 nm)	UV/LCD/COB (wavelength 405 nm)	UV LED/LCD (wavelength 405 nm)
Print Speed	10–50 mm/h (depends on layer thickness and materials)	30–70 mm/h	20–36 mm/h

Abbreviations: LED = Light-Emitting Diode; LCD = Liquid Crystal Display; UV = ultraviolet; COB = Chip On Board (continuous light source).

**Table 2:** The mean value (SD) of linear measurements (mm) of the test bodies printed with 3 different 3D printers for each time point (T0, T1, T2, and T3).

Printers		AccuFab				Elegoo				Zortrax			
Time point		T0 (mm)	T1 (mm)	T2 (mm)	T3 (mm)	T0 (mm)	T1 (mm)	T2 (mm)	T3 (mm)	T0 (mm)	T1 (mm)	T2 (mm)	T3 (mm)
Mean value		12.199 (7.94)	12.222 (7.95)	12.343 (8.00)	12.250 (7.95)	12.093 (7.98)	12.096 (7.98)	12.203 (7.99)	12.138 (8.00)	12.073 (7.94)	12.183 (7.94)	12.266 (7.96)	12.202 (7.92)
Test body shape 1	Y	23.904 (0.14)	23.961 (0.15)	24.149 (0.02)	23.967 (0.17)	23.791 (0.14)	23.794 (0.13)	23.935 (0.14)	23.933 (0.03)	23.825 (0.12)	23.881 (0.07)	23.995 (0.04)	23.817 (0.03)
	X	17.980 (0.09)	17.983 (0.12)	18.133 (0.01)	18.022 (0.10)	17.886 (0.06)	17.891 (0.07)	18.021 (0.08)	17.927 (0.04)	17.860 (0.10)	17.865 (0.02)	17.993 (0.05)	17.941 (0.08)
Test body shape 2	Y	19.908 (0.14)	19.954 (0.17)	20.139 (0.03)	20.001 (0.46)	19.855 (0.09)	19.861 (0.09)	19.990 (0.13)	19.945 (0.04)	19.623 (0.33)	19.870 (0.09)	19.962 (0.07)	19.884 (0.11)
	X	14.959 (0.07)	14.993 (0.12)	15.083 (0.05)	14.985 (0.09)	14.914 (0.04)	14.909 (0.04)	14.975 (0.05)	14.899 (0.05)	14.875 (0.10)	14.909 (0.06)	14.917 (0.06)	14.883 (0.04)
Test body shape 3	Y	9.929 (0.08)	9.913 (0.08)	10.048 (0.01)	9.959 (0.09)	9.931 (0.04)	9.926 (0.03)	10.011 (0.05)	9.911 (0.03)	9.929 (0.05)	10.111 (0.16)	10.351 (0.08)	10.244 (0.11)
	X	7.964 (0.06)	8.002 (0.06)	8.151 (0.26)	8.087 (0.02)	7.848 (0.04)	7.843 (0.04)	7.918 (0.05)	7.869 (0.04)	7.859 (0.04)	8.091 (0.09)	8.124 (0.09)	8.087 (0.08)
Test body shape 4	Thickness	1.941 (0.05)	1.965 (0.04)	1.993 (0.01)	1.973 (0.04)	1.717 (0.09)	1.729 (0.13)	1.865 (0.05)	1.776 (0.10)	1.764 (0.11)	1.855 (0.05)	1.878 (0.05)	1.858 (0.05)
Test body shape 5	Thickness	1.005 (0.04)	1.004 (0.03)	1.045 (0.01)	1.011 (0.04)	0.803 (0.06)	0.819 (0.07)	0.906 (0.06)	0.845 (0.04)	0.846 (0.05)	0.880 (0.06)	0.904 (0.07)	0.900 (0.07)

Abbreviations: T0 = Effect of printing and post-processing. T1 = Effect of storage time after printing and post-processing. T2 = Effect of steam sterilization. T3 = Effect of storage time after steam sterilization.

## Data Availability

The raw data are not readily available because they are part of an ongoing study. Requests for access should be directed to the corresponding author.
